# Meta-analysis reveals that grain quality is improved in ratoon season crop compared with main crop

**DOI:** 10.3389/fpls.2025.1604686

**Published:** 2025-10-22

**Authors:** Xuelian Yang, Zhijie Ruan, Kezhi Chen, Shiting Gu, Yuchao Wang, Qiang Cai, Li-Ang Yao, Mohammad Reza Boorboori, Arafat Yasir, Juthamas Chaiwanon, Kanako Bessho-Uehara, Kihye Shin, Yunfei Wu, Wenfei Wang, Wenxiong Lin

**Affiliations:** ^1^ Fujian Provincial Key Laboratory of Agroecological Processing and Safety Monitoring, College of Juncao Science and Ecology, Fujian Agriculture and Forestry University (FAFU), Fuzhou, China; ^2^ Key Laboratory of Crop Ecology and Molecular Physiology (FAFU), Fujian Province University, Fuzhou, China; ^3^ College of Environment and Surveying and Mapping Engineering, Suzhou University, Suzhou, China; ^4^ State Key Laboratory of Desert and Oasis Ecology, Xinjiang Institute of Ecology and Geography, Chinese Academy of Sciences, Urumqi, China; ^5^ Center of Excellence in Environment and Plant Physiology, Department of Botany, Faculty of Science, Chulalongkorn University, Bangkok, Thailand; ^6^ Graduate School of Life Sciences, Tohoku University, Sendai, Japan; ^7^ Department of Microbiology and Immunology, Jeju National University College of Medicine, Jeju, Republic of Korea; ^8^ Jiangsu Key Laboratory of Crop Genetics and Physiology/Co-Innovation Center for Modern Production Technology of Grain Crops/Joint International Research Laboratory of Agriculture andAgri-Product Safety, Yangzhou University, Yangzhou, China

**Keywords:** ratoon season crop (RC), main crop (MC), rice quality, meta-analysis, milling traits, chalkiness

## Abstract

Rice ratooning cultivation refers to secondary production from the stubble left after the harvest of the main crop (MC). Besides providing additional yield, ratooning is known to affect grain quality. Here, we conducted a meta-analysis of grain quality traits between ratoon season crop (RC) and MC. The results showed that the overall grain quality of RC was higher than that of MC. Ratooning improved milling traits by increasing the milled rice rate (MRR) and head rice rate (HRR) and enhanced grain appearance by reducing chalkiness. Furthermore, ratooning had a positive impact on alkali spreading value (ASV) and amylose content (AC) but a negative influence on gel consistency (GC), which markedly affects cooking and sensory quality. Subgroup analysis showed that stubble height influenced the AC of RC, while planting region was a major factor regulating most grain properties. Meta-regression analysis suggested that latitude, precipitation, and temperature played important roles in rice quality, particularly in relation to milling parameters. In addition, we compared the grain quality of RC and the late-season crop (LC) with synchronized heading time. Negative trends were observed in brown rice rate (BRR) and protein content (PC), while planting region and rice variety were revealed as factors influencing chalkiness and HRR. Overall, our findings indicate that ratooning has positive impacts on grain quality and uncover the relationships between environmental and agronomic factors and their effects on quality traits, which will lay the foundation for future breeding strategies and optimize cultivation management across growth regions.

## Introduction

Ratoon rice, a second rice production system generated from the stubble left after harvesting the main crop (MC), is considered an ideal cropping system in regions where light and temperature resources are sufficient for one seasonal crop but insufficient for two (IRRI, 1988). Rice ratooning is an ancient cultivation technique, with a history of more than 1,700 years since the West Jin Dynasty (AD 265–316) in China (Guo, 1993). It is now fairly widespread in many countries, including Japan, Korea, India, Thailand, Vietnam, the Philippines, Indonesia, the United States, and Nigeria (IRRI, 1988; Calendacion et al., 1992; Munda et al., 2009; Sanni et al., 2009; Wang et al., 2020b; Deng et al., 2021).

The main advantages of rice ratooning are promising high grain yield, high economic efficiency, and environmental friendliness (Lin et al., 2015). More recently, several studies have reported another potential advantage—grain quality of the ratoon crop (RC) may be better than that of the MC (Alizadeh and Habibi, 2016; Huang et al., 2020; Wang et al., 2020b). However, other studies found that the cooking quality of rice decreased after ratooning (Shin et al., 2015; Wang et al., 2020a). Thus, the impact of ratooning on grain quality appears to vary among studies.

Rice quality is determined by multiple traits, and quality perception varies across consumers and countries (Custodio et al., 2019). Several traits are widely used to evaluate rice quality, including milling properties, appearance, nutritional value, and cooking and sensory quality (Fitzgerald et al., 2009). Milling quality refers to the final yield of edible rice and the proportion of unbroken kernels, which is mainly evaluated by brown rice rate (BRR), milled rice rate (MRR), and head rice rate (HRR). Appearance is a crucial property after milling, commonly assessed by chalky rice rate (CRR), chalkiness degree (CD), and length–width ratio (LWR). Previous studies have reported significant reductions in chalkiness degree and CRR in ratoon-season grains (Alizadeh and Habibi, 2016; Cai et al., 2019; Lin et al., 2022). Cooking and sensory quality are usually predicted by alkali spreading value (ASV), gel consistency (GC), and amylose content (AC). Some studies showed that ratoon-season grains had higher AC and significantly lower transition gelatinization temperatures (Shin et al., 2015; Kuang et al., 2021). In addition, consumers are concerned about nutritional value, which mainly refers to the amounts of protein, vitamins, minerals, and lipids in the grain.

Rice quality can be influenced by cultivar, management practices, and environmental conditions during grain filling. Ratooning ability, yield, and quality vary among different types of varieties (Negalur et al., 2017). Recent studies have revealed that environmental factors, including precipitation, temperature, fertilizer application, and biotic and abiotic stresses, play key roles in ratooning production and quality (Wang et al., 2020b). For example, the cooking and eating quality of ratoon rice was higher in lower-latitude regions (Kuang et al., 2021). Crop management practices such as stubble height, irrigation, and nitrogen fertilization also affect ratoon rice (Jones, 1993; Chen et al., 2021; Yang et al., 2021). However, how these factors influence final grain quality remains largely unclear.

Meta-analysis, a quantitative synthesis of research results, allows assessment of overall trends (Gurevitch et al., 2018). Given the variability in findings regarding ratooning and grain quality, we used meta-analysis to integrate results across studies. We summarized and interpreted 59 studies to assess overall rice quality and quantify the effects of ratooning. Our results suggest that ratoon-season grain quality is generally better than that of the MC, with improvements in milling properties, reductions in chalkiness, and modifications in cooking and nutritional traits. Subgroup and meta-regression analyses further revealed relationships between grain quality and cultivar, management, and environmental factors. These findings provide a basis for future breeding strategies and optimization of cultivation management across different growth regions.

## Materials and methods

### Definitions of comparison groups

Two comparisons were conducted in this meta-analysis: (a) RC grain quality vs. MC grain quality, and (b) RC grain quality vs. late-season crop (LC) grain quality.

After harvesting the MC, the second rice crop generated from the stubble was defined as the RC. The LC refers to the late-season main crop with heading time synchronized with the RC, meaning that grains from both crops were obtained under the same weather conditions.

### Literature selection

A systematic search was conducted to identify peer-reviewed studies describing the grain quality of ratoon rice, the main crop, and the late season crop. Ten grain quality parameters, including brown rice rate, milled rice rate, head rice rate, chalky rice rate, chalkiness degree, length-width ratio, alkali spreading value, gel consistency, amylose content, and protein content, were selected to conduct the meta-analysis.

Articles were collected through PubMed (https://pubmed.ncbi.nlm.nih.gov), Google Scholar (https://scholar.google.com), Web of Science (https://www.webofknowledge.com), Science Direct (https://www.sciencedirect.com), SpringerLink (https://link.springer.com), Wiley Online Library (https://onlinelibrary.wiley.com/), Scientific Information Database (https://www.sid.ir/), Taylor & Francis (https://www.tandfonline.com/), CNKI database (https://www.cnki.net/), Wanfang database (https://www.wanfangdata.com.cn/index.html), CQVIP database (http://www.cqvip.com/), Huayi Database (http://www.airitilibrary.cn/), CiNii research (https://cir.nii.ac.jp/) and AGRIS (https://agris.fao.org). Data collection was restricted to field studies; studies conducted in laboratories, greenhouses, or pots. The following four selection criteria were applied:

Criterion 1: We included studies that recorded rice quality data for either “ratoon rice vs. main crop” or “ratoon rice vs. late-season main crop.” Studies reporting only general conclusions on rice quality without specific index data were excluded.
Criterion 2: The rice quality index data that we accepted only contained brown rice rate, milled rice rate, head rice rate, chalky grain rate, chalkiness degree, length-width ratio, alkali spreading value, gel consistency, amylose content, and protein content. Articles with grain length and width data, which allowed us to calculate the length-width ratio, were included.
Criterion 3: Studies with only one dataset and those on overwintering cultivated rice (a specific cropping practice in the low latitude zone with adapted local varieties) were excluded.
Criterion 4: Duplicate datasets from the same study were excluded after careful comparison.

Study selection followed the PRISMA protocol (Liberati et al., 2009; Moher et al., 2009) (**Supplementary Figure S1**). From August 2021 to May 2023, we used combinations of keywords to search for relevant literature. The keywords included (“ratoon rice” OR “ratooning” OR “ratoon” OR “double season rice” OR “double cropping”) AND (“quality” OR “appearance” OR “milling properties” OR “milling” OR “cooking and sensory quality” OR “cooking” OR “sensory” OR “nutrient” OR “nutrition” OR “brown rice” OR “milled rice” OR “head rice” OR “chalky” OR “chalkiness” OR “milk white grain” OR “chalk white” OR “floury endosperm” OR “length-width ratio” OR “alkali spreading value” OR “gel consistency” OR “amylose content” OR “amylose” OR “protein content” OR “protein”) without restriction on publication year. A total of 1,479 articles were initially identified. With the help of international researchers, 28 additional foreign-language articles were selected. After excluding duplicates, 1,422 articles remained for screening. During the second screening, 246 papers were retained after reviewing titles, keywords, and abstracts. The full texts of these papers were then assessed for suitability. Finally, 56 articles were included for RC vs. MC comparison, and 9 articles for RC vs. LC comparison (see **Supplementary Database**).

**Figure 1:**
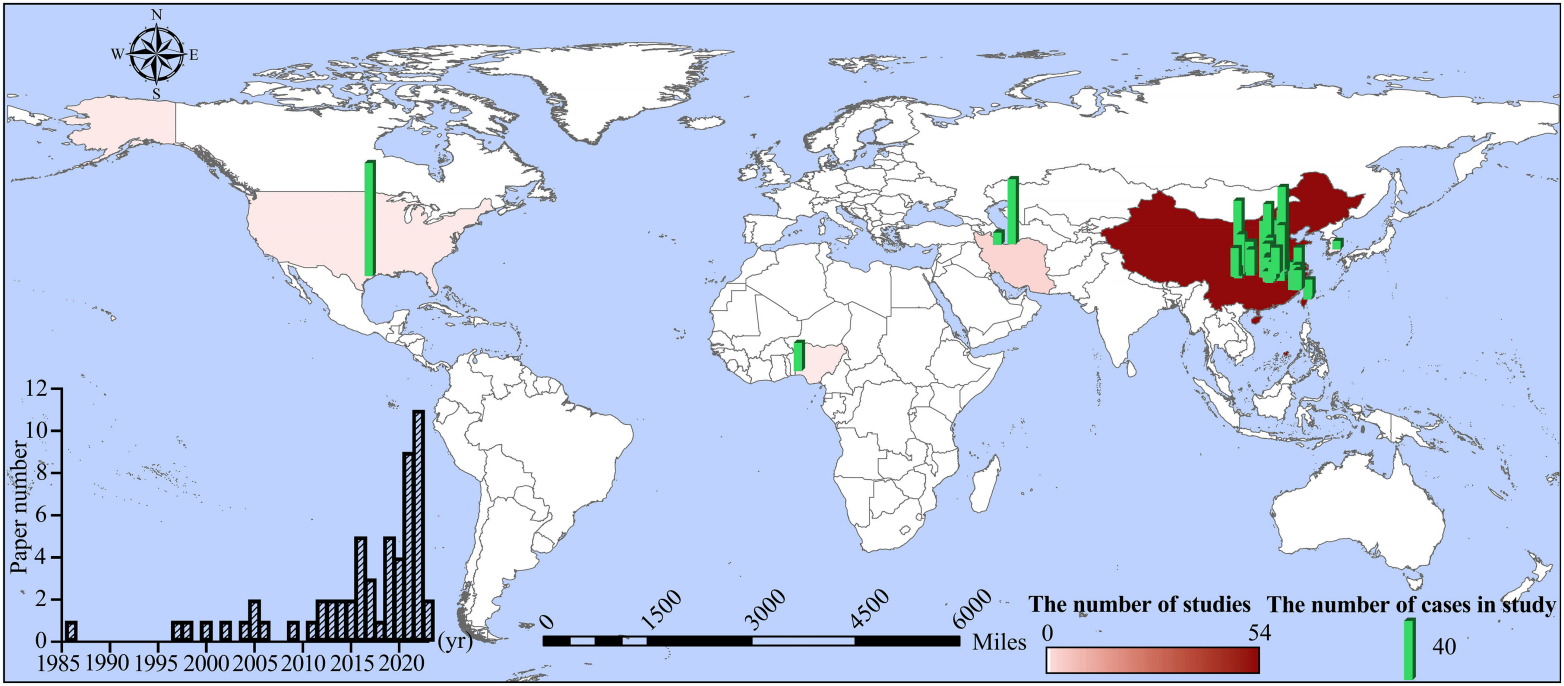
Distribution of reviewed studies related to grain quality of ratoon rice. The world map shows ratoon rice studies located around the world. Country color indicates the number of studies, and column height indicates the number of experimental cases in each study. The inset column plot in the lower left shows the number of reviewed articles per year (from 1986 to 2023). The horizontal bar with gradient red colors shows the number of studies. The vertical bar shows the number of cases in each study. For detailed figure information, see **Supplementary Data** and source data.

All data were integrated into a world map (**Figure 1**), generated using resources from the Resource and Environment Science and Data Center in China (https://www.resdc.cn/Default.aspx).

### Risk of bias assessment

The Cochrane system was used to assess the risk of bias in all studies included in the meta-analysis. The assessment was conducted in six areas: “Sequence generation (selection bias),” “Blinding of participants (performance bias),” “Blinding of outcome assessment (detection bias),” “Incomplete outcome data (attrition bias),” “Selective outcome reporting (reporting bias),” and “Other potential threats to validity (other bias),” to detect deficiencies in study design, implementation, analysis, and reporting integrity (Source Data **Supplementary Figure S2B**). Due to the objectivity of the agricultural experiment process, in the ‘Selection bias’, we only adopted the assessment of ‘‘Sequence generation’’ while excluding ‘‘Allocation concealment’’ (Higgins et al., 2017). For performance bias, the evaluation criterion was whether the experimental field areas were managed uniformly to prevent staff from knowing which interventions the experimental subjects received. For detection bias, the criterion was whether the study provided a detailed explanation of the rice quality testing methods and strictly adhered to them to avoid testing bias caused by interventions from testers. Following the guidance of the Cochrane Handbook for Systematic Reviews of Interventions, we classified and evaluated each article as having “low risk of bias,” “unclear risk,” or “high risk of bias” (Source Data **Supplementary Figure S2A**) (Saric et al., 2019). Figures were generated using the “ggplot” package in R software (version 4.3.1).

### Study quality assessment

Because no standardized system exists for evaluating the quality of the studies included, we applied “21 quality checklist questions” concerning the rationality of the experimental design and the strength of evidence for improvement of rice quality indicators through ratooning (Su et al., 2021) (Source Data **Supplementary Figure S3**).

According to the classification criteria of Su et al., study quality was categorized as very strong evidence (score >75%), strong evidence (score 50%–74%), moderate evidence (score 25%–49%), or weak evidence (score <24%). For this meta-analysis, we included only studies with very strong (score >75%) and strong (score 50%–74%) evidence.

### Meta-analysis

From each study, we extracted rice quality data, including BRR, MRR, HRR, CRR, CD, LWR, ASV, GC, AC, and PC. For the “RC vs. MC” comparison, data were included only when they came from the same year, in the same experimental field, and with the same variety. For the “RC vs. LC” comparison, data were included only when matched for the same variety and heading time.

The standardized mean difference (SMD; Cohen’s d) was used as the effect size, calculated as the difference in means between two groups divided by the standard deviation. When comparing RC with MC or LC, the SMD value reflected whether grain quality increased or decreased (Lachenbruch, 1989). Meta-analysis was performed using a random-effects model with the constrained maximum likelihood (CML) method. The 95% confidence interval (CI) in the forest plots indicated significant positive or negative changes (Nakagawa and Cuthill, 2007; Borenstein et al., 2010). Heterogeneity was tested using the Q test and quantified with the I² statistic; generally, I² > 50% indicated significant heterogeneity among studies (Hedges and Olkin, 1985; Higgins et al., 2003). All analyses were performed in Stata version 16, following published guidelines for biological meta-analysis (Koricheva et al., 2013; Chaimani et al., 2014; Nakagawa et al., 2017).

Grain quality was evaluated according to the Chinese national standard for high-quality paddy (GB/T 17891-2017) (**Supplementary Database**). Grains were first classified as either *indica*-type rice or *japonica*-type rice. *Indica* rice, *indica*-type hybrids, and *indica*–*japonica* hybrids were evaluated under the “indica-type rice” standard, while *japonica* rice and *japonica*-type hybrids were grouped under “*japonica*-type rice.” The *indica* group was further subdivided into “long grain”, “medium grain”, and short-grain categories based on reported grain length.

Grain quality was assessed using three parameters: HRR, CD, and AC. According to the standard, grain was scored as Grade 1 (score = 4), Grade 2 (score = 3), or Grade 3 (score = 2). If quality was lower than Grade 3, it was classified as Grade 4 (score = 1).

### Subgroup analysis

Subgroup analyses were conducted for (a) stubble heights after the main rice harvest (more than 20 cm and below 20 cm), (b) rice varieties (*indica* rice, *japonica* rice, and hybrid rice), and (c) the geographical regions of study (e.g., South China Plain Hilly, Jiangnan Hilly Plain, Middle-lower Yangtze Plain, Sichuan Basin, Northern Iran, southern United States, Korea). These subgroup analyses were used to determine the source of heterogeneity among different studies by calculating effect sizes within each subgroup and analyzing their differences (Thompson and Higgins, 2002).

### Meta-regression analysis

Factors with continuous data were used as variables for meta-regression analysis, including (a) latitude of the experimental area (all are in the Northern Hemisphere), meteorological factors in the grain-filling period (two months), including (b) average precipitation per day (ΔPrecipitation), (c) average temperature per day (ΔTavg), (d) nitrogen (N) application, (e) planting density, (f) study year, and (g) average global surface solar radiation per year compared RC and MC. The application of nitrogen fertilizer described in the article during the main crop cutting period was converted into total nitrogen amount as “kilograms per hectare”. Planting density was measured as “thousands of hills per hectare,” which was recalculated according to the data from each study. The year of study was the trial time described in each study, not the article publication time. Global historical climate datasets are provided by the “National Centers for Environmental Information” (https://www.ncei.noaa.gov/). Global surface solar radiation datasets are provided by the “National Tibetan Plateau Data Center” (http://data.tpdc.ac.cn) and “Geographic remote sensing ecological network” platform (www.gisrs.cn). All the data were extracted from the articles to be categorized and recalculated. Bubble plots were used to present the results of regression analyses on correlations between effect sizes and variables (Cameron and Windmeijer, 1997).

### Tests of publication bias

Egger’s regression was performed to evaluate publication bias and presented as funnel plots, with parameters set as default (Egger et al., 1997).

## Results

### General literature description

Different methods were used to search articles about grain quality of MC, RC, and LC, in which rice heading time was synchronized with ratoon rice. A total of 1,507 articles from multiple databases were found to be relevant to rice grain quality (**Supplementary Figure S1**). After removing papers with duplicated data (N = 85) and unqualified data (N = 1,361), 59 articles met our criteria and were selected for analysis (**Supplementary Database** “list of publication”). The studies comparing RC and MC numbered 56, while studies referring to RC and LC numbered only 9 (**Supplementary Database** “RC vs MC” and “RC vs LC”). Six studies included grain quality data for RC, MC, and LC. The PRISMA flow diagram for the studies selected and included in our meta-analysis is shown in **Supplementary Figure S1**. The geographical distribution of the studies spanned 5 countries: China (53 studies), Iran (3 studies), Korea (1 study), the United States (1 study), and Nigeria (1 study) (**Figure 1**). The first article comparing grain quality between RC and MC was published in China in 1986. A rapid increase in publications occurred after 2010 (**Figure 1**, lower left chart), as governments and researchers devoted greater attention to ratoon rice over the past ten years.

The risk of bias and study quality assessments were performed to evaluate study reliability. According to the *Cochrane Handbook* for systematic reviews of interventions, six factors were assessed to qualify risk of bias (**Supplementary Figure S2**). The results showed that all assessment factors represented low risk of bias. Study quality was further assessed using “21 quality checklist questions.” Based on the scores, the studies included in this analysis provided strong evidence (>60%) (**Supplementary Figure S3**).

All detailed figure information and values are provided in the **Supplementary Material** (“source data”).

### Effects of ratooning on the grain quality

Ten parameters from four primary quality traits were investigated in this study, covering milling properties (BRR, MRR, HRR), appearance properties (CRR, CD, LWR), cooking and sensory quality (ASV, GC, AC), and nutritional value (PC). All 10 grain traits were compared between RC and MC and collected for meta-analysis (using standardized mean difference (SMD; Cohen’s d) as the effect size (ES)). In total, 1,138 observations met the inclusion criteria (**Supplementary Database** “RC vs MC” and “RC vs LC”).

**Figure 2:**
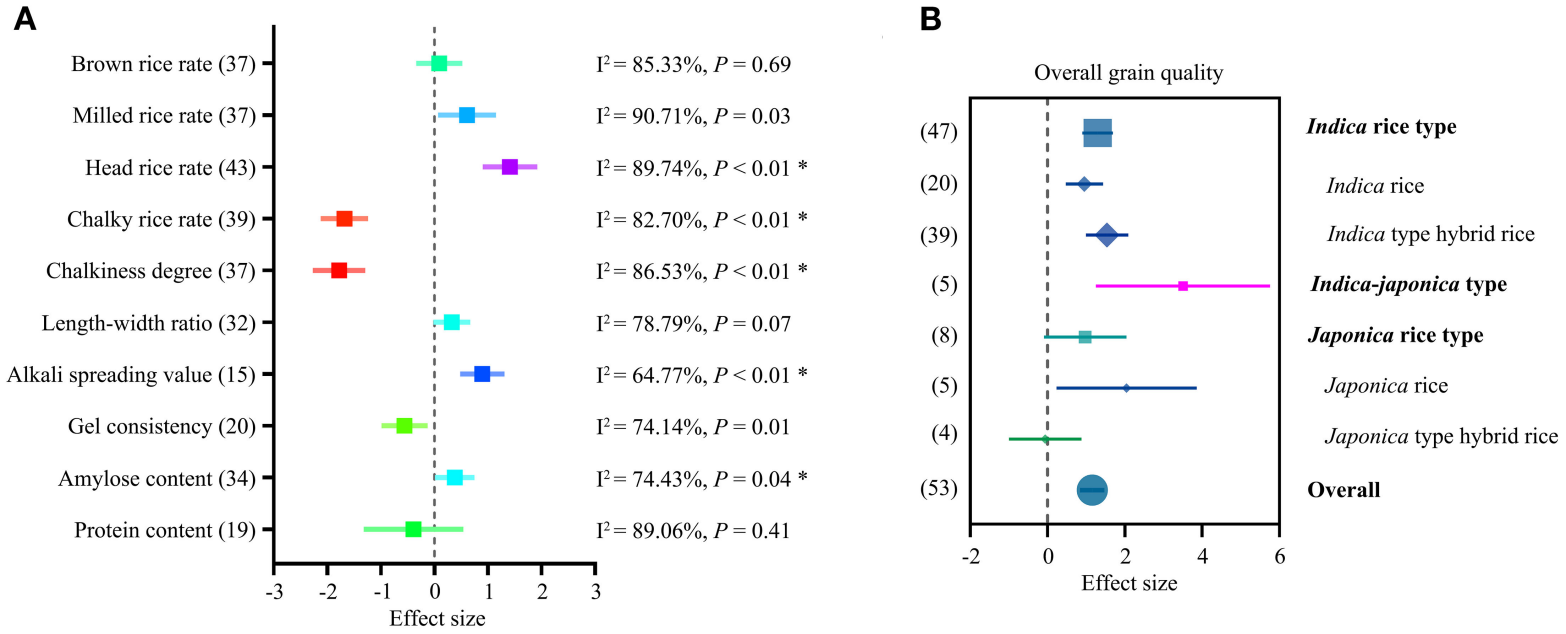
Mean effect sizes (ES) for the grain quality compared RC to MC. **(A)** ES of rice quality traits by ratooning (RC vs. MC). Heterogeneity value (I²) and the *P* value are presented on the right. Asterisks indicate that publication bias was detected. **(B)** ES on overall grain quality comparing RC to MC. Grain quality was evaluated by the Chinese national standard of high-quality paddy (GB/T 17891-2017). ES is plotted as SMD (Cohen’s d). Bars around the means denote 95% CIs. The bracketed numbers in A and B represent the number of accepted studies.

Compared with MC, the milling properties MRR and HRR of RC increased, with ES values of 0.61 (95% CI = 0.07 to 1.15) and 1.41 (95% CI = 0.90 to 1.92), respectively, which was considered a significant improvement (**Figure 2A**). For appearance traits, CRR and CD showed negative trends in RC compared with MC, with ES values of –1.68 (95% CIs = -2.12 to -1.24) and -1.78 (95% CIs = -2.27 to -1.29), respectively (**Figure 2A**), indicating improved appearance quality in ratoon rice.

Ratooning also had positive effects on ASV (ES = 0.90, 95% CI = 0.48 to 1.31) and AC (ES = 0.39, 95% CI = 0.02 to 0.76) (**Figure 2A**). In contrast, GC—an important parameter of eating quality—was reduced by ratooning (ES = –0.59, 95% CI = –0.97 to –0.11).

The overall grain quality was also evaluated according to the Chinese national standard for high-quality paddy (GB/T 17891-2017) (**Supplementary Database**), which is widely used in Chinese rice research and markets. Taking into account cooking and sensory parameters, as well as milling and appearance traits, rice grains were rated into four grades. Comparison of RC and MC grades showed that overall grain quality improved after ratooning, in both *indica* and *japonica* rice types (**Figure 2B**).

It is noteworthy that the funnel plots of five grain traits—HRR, CRR, CD, ASV, and AC—were asymmetric (**Supplementary Figure S4**), indicating potential publication bias in the relevant literature, marked with stars in **Figure 2A**. All potential publication biases were corrected using the trim-and-fill method, and the results did not change the conclusions (**Supplementary Figure S4**, **Supplementary Table 4**).

Taken together, comparison of grain qualities between RC and MC showed that ratooning improved overall grain quality across multiple traits.

### Subgroup analysis of the categorical factors

**Figure 3:**
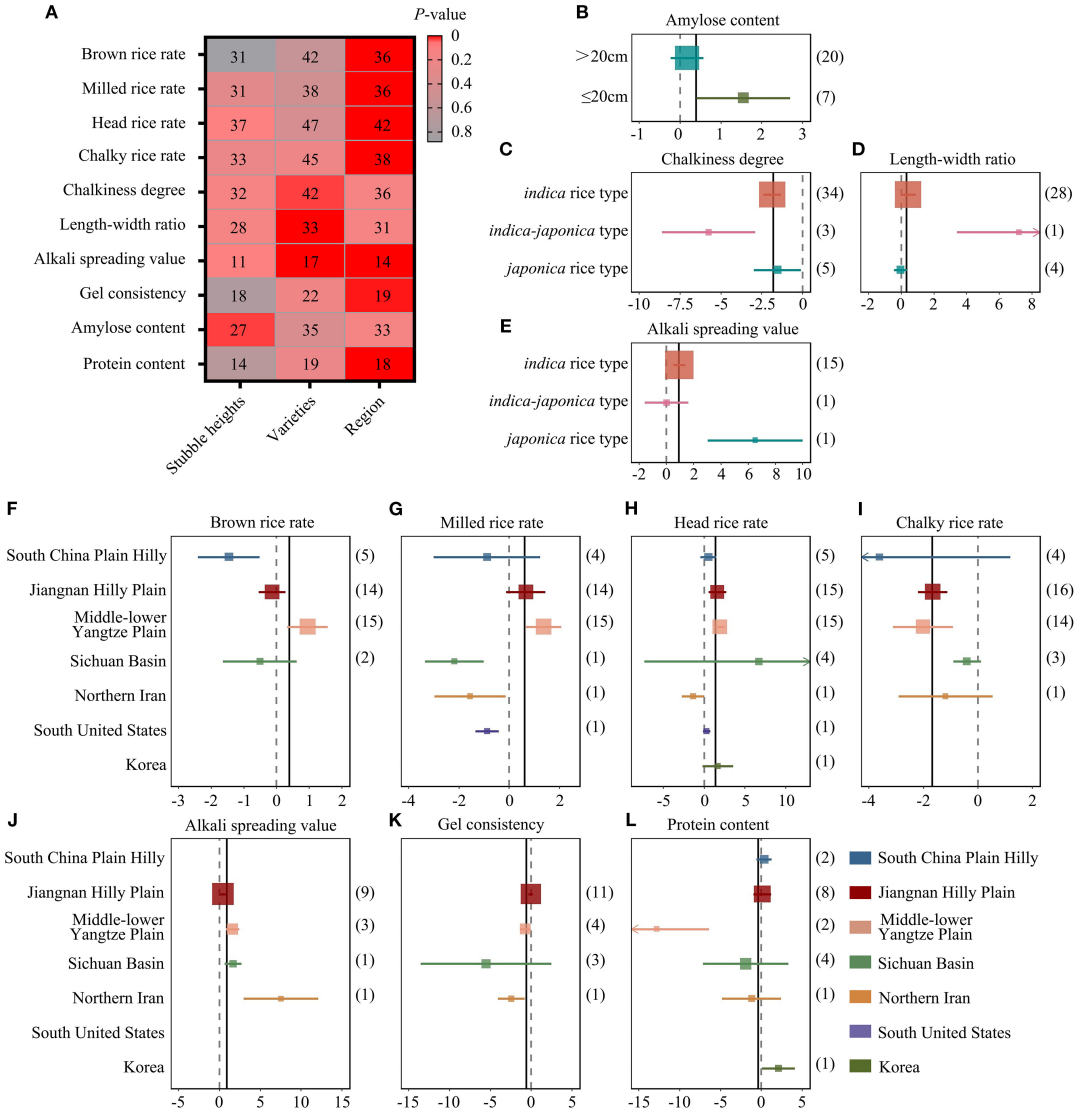
Subgroup analysis of categorical factors. **(A)** Heatmaps of correlations between grain quality traits and categorical factors (stubble heights, rice varieties, and study region). Gradient red color indicates *P*-values of correlations. Numbers in boxes represent the number of research cases included in the analysis. **(B)** Effects of stubble height on AC. **(C–E)** Effects of rice varieties on CD, LWR, and ASV. **(F–L)**. Effects of geographical regions on grain quality parameters [**(F)** BRR; **(G)** MRR; **(H)** HRR; **(I)** CRR; **(J)** ASV; **(K)** GC; **(L)** PC]. In **(B–L)**, ES is plotted as SMD (Cohen’s d) comparing RC to MC. Bars around the means denote 95% CIs. For milling properties, if the means fall on the positive side and do not intersect with zero, BRR, MRR, and HRR are considered to increase relative to MC; the opposite applies if means fall on the negative side of the forest plot. For appearance properties (CD and CRR), negative means indicate decreases in RC compared with MC. All seven analyzed regions are shown in different colors. The vertical black line indicates the pooled ES of all categorical factors. Bracketed numbers represent the numbers of accepted study cases. For Q statistic tests, see **Supplementary Table 1**. For detailed SMD values, (Cohen’s d), see “Source data”.

Significant heterogeneity (I^2^ ≥50%) appeared among studies in all grain traits (**Figure 2A**), suggesting that various factors may contribute to grain quality. Thus, to identify the potential influencing factors on the heterogeneity of the pooled ES, the following subgroup and meta-regression analyses were performed. Stubble height, rice variety, and study region were analyzed as categorical moderators for their contributions to grain quality change between RC and MC (**Figure 3A**).

Stubble height was divided into two groups: less than 20 cm and more than 20 cm. Our subgroup analysis showed that ratooning significantly promoted AC in the “less than 20 cm” group but not in the “more than 20 cm” group (**Figure 3B**). The influence of ratooning on CD, LWR, and ASV varied between cultivars (**Figures 3C–E**). *Japonica*, *indica*, and hybrid rice are three major planting cultivars today. Our subgroup analysis showed that CD decreased most in hybrid rice, although all three cultivars had lower CD in RC compared with MC (**Figure 3C**). Indica grain also showed slight increases in LWR (ES = 0.41, 95% CI = –0.04 to 0.87) and ASV (ES = 0.93, 95% CI = 0.49 to 1.37) (**Figures 3D, E**).

Planting region was found to be a major influencing factor on grain quality parameters, including BRR, MRR, HRR, CRR, ASV, GC, and PC (**Figure 3A**). Based on geographical and weather conditions, all tested cases were divided into six planting regions: South China Plain Hilly, Jiangnan Hilly Plain, Middle–Lower Yangtze Plain, Sichuan Basin, Northern Iran, southern United States, and Korea (**Figures 3F–L**). With ratooning, milling properties (BRR and MRR) especially increased in the Middle–Lower Yangtze Plain but decreased in South China (**Figures 3F, G**). HRR of ratoon rice was promoted in most parts of China (**Figure 3H**).

The effects on appearance parameters also varied across geographical regions. Significantly negative trends for CRR were observed in all study cases from China (**Figure 3I**). For other traits such as ASV, GC, and PC, most studies were conducted in the Jiangnan Hilly Plain; however, no obvious changes were observed after ratooning (**Figures 3J–L**). In other planting regions, fewer cases were available, making it difficult to draw firm conclusions about grain quality changes.

### Regression analysis of the continuous factors

**Figure 4:**
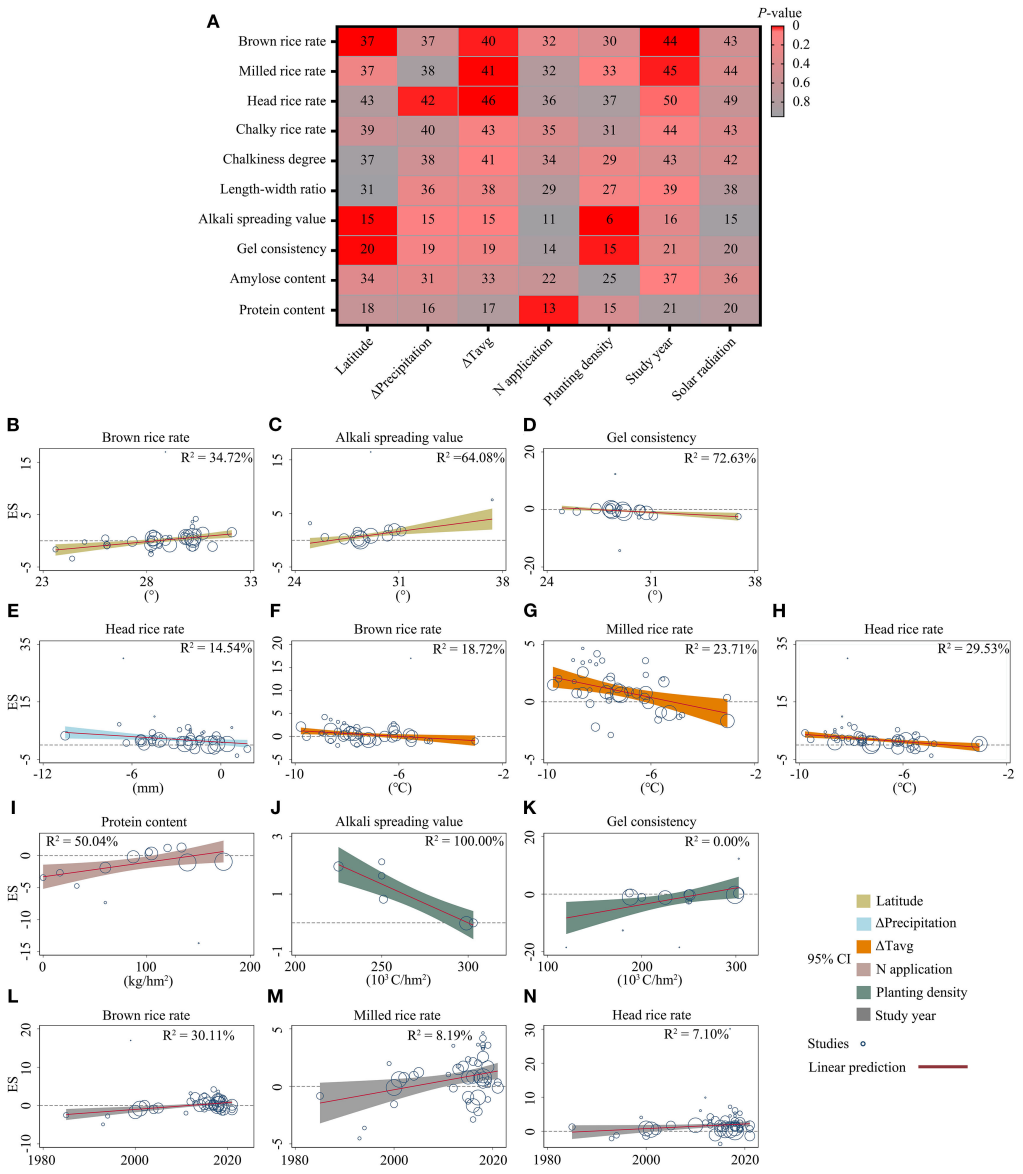
Regression analysis of continuous factors. **(A)** Heatmaps of correlations between grain quality traits and continuous factors (latitude, the difference of “average precipitation per day” compared RC and MC (Δprecipitation), the difference of “average temperature per day” compared RC and MC (ΔTavg), application of nitrogen (N) fertilizer, planting density, study year, and solar radiation). Red color indicates *P*-values. Numbers in boxes represent the number of research cases included in the analysis. **(B–N)** Bubble plots showing predicted trends of continuous factors on grain quality traits. Bubble sizes reflect sample sizes in individual studies. R² values for meta-regression analysis are shown in **Supplementary Table 3**.

Besides the above categorical factors, associations between grain trait trends and six hypothesized continuous factors were estimated, including latitude, the difference in “average precipitation per day” (Δprecipitation), the difference in “average temperature per day” (ΔTavg), application of nitrogen (N) fertilizer in the studies, planting density, study year, and solar radiation between RC and MC (**Figure 4A**). All collected studies were conducted in the Northern Hemisphere, ranging from N 22° to N 42°.

Meta-regression analysis revealed that latitude had a moderate positive relationship with BRR and ASV (**Figures 4B, C**) but a negative relationship with GC (**Figure 4D**). HRR was positively correlated with a decrease in precipitation in the ratoon season (**Figure 4E**), while all three milling properties (BRR, MRR, and HRR) were positively correlated with a decrease in average temperature in the ratoon season (**Figures 4F–H**). For N fertilizer application, PC was positively related to the amount of nitrogen applied (**Figure 4I**). Planting density was also found to have an absolute negative relationship with ASV (R^2^ = 100%) and a clearly positive relationship with GC (**Figures 4J, K**). With increasing study years, positive correlations were observed for all milling properties, including BRR, MRR, and HRR (**Figures 4L–N**).

### Comparison of grain qualities between RC and LC

Previous studies suggested that the improvement of rice quality in RC was mainly due to relatively lower temperatures during the filling period compared with MC (Liu et al., 2002), which was also supported by our subgroup analysis of ΔTavg. To further investigate the potential factors affecting grain quality, we performed a meta-analysis comparing RC and LC, since LC was grown under the same environmental conditions as ratoon rice. In total, 112 observations met our criteria.

**Figure 5:**
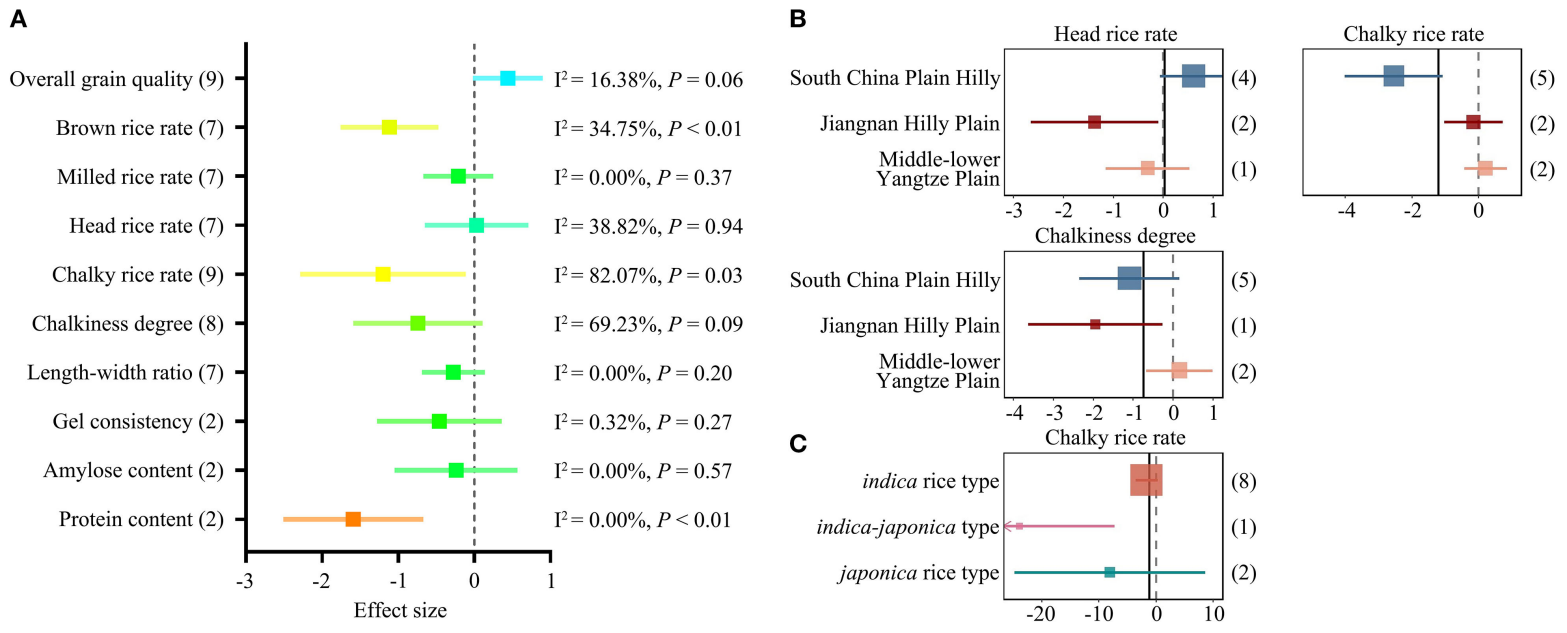
Mean ES for the grain quality compared RC and LC. **(A)** ES distribution is plotted as SMD (Cohen’s d) comparing RC to LC. Bracketed numbers represent the number of accepted study cases. Heterogeneity value (I^2^) and the *P* value are presented on the right. **(B)** Subgroup analysis of planting regions for grain quality traits comparing RC to LC. **(C)** Subgroup analysis of rice cultivar varieties for CRR comparing RC to LC. The vertical black line in **(B, C)** indicates the pooled ES of all categorical factors. Bars around the means denote 95% CIs. For Q statistic tests, see **Supplementary Table 2**.

Compared with LC, the appearance properties of RC were also improved. CRR decreased with an ES of –1.20 (95% CI = –2.29 to –0.11). BRR and PC also decreased, with ES values of -1.11 (95% CIs = -1.76 to -0.47) and -1.59 (95% CIs = -2.51 to -0.67), respectively (**Figure 5A**). Other grain traits showed no significant differences between RC and LC (P>0.05).

Considering the heterogeneity among studies, subgroup analysis was also performed. Ratooning increased HRR especially in South China but decreased it in Central China (**Figure 5B**). In addition, CRR decreased only in South China during the ratoon season, while no obvious differences were observed in Central China (**Figure 5B**). For CD, some decreases were observed in South China and the Jiangnan Hilly Plain. Furthermore, the effect on CRR varied between cultivars: CRR decreased in hybrid rice but not in *indica* cultivars (**Figure 5C**).

## Discussion

### Impacts of ratooning to grain quality

In our meta-analysis, milling properties were dramatically improved in RC compared with MC, especially in China. The observed increase in HRR is consistent with the decline in chalkiness, as chalky kernels are more susceptible to breakage during milling (Siebenmorgen et al., 2013). Chalkiness is a complex trait attributed to both genetic and environmental factors, particularly high temperature (Nevame et al., 2018). Ratooning clearly decreased the chalky rice rate, supported by studies in Iran and China. Interestingly, research from the Sichuan Basin reported minimal changes in CD, potentially attributable to localized climate conditions, cultivar selection, or management practices.

Based on the viscosity of 4.4% milled-rice paste, rice can be divided into three categories: soft (>60 mm), adhesive (40–60 mm), and hard (<40 mm) (Zhang et al., 2020). A significant decrease was found in GC, which was negatively correlated with increasing latitude and positively correlated with planting density. This aligns with Kuang et al. (2021), who observed a 15% reduction in GC for ratoon rice at higher latitudes (Xinyang, 32.1°N) compared with lower-latitude sites (Changsha, 28.2°N; Zhaoqing, 23.0°N).

ASV is generally considered to represent the gelatinization temperature, which is negatively correlated with the cooking temperature of rice (Graham, 2002). In contrast to the negative impact on GC, ASV increased in the ratoon season, consistent with previous studies showing that ASV is negatively related to GC in a recombinant inbred line population derived from a *japonica*/*indica* cross (Wang et al., 2007; Lu et al., 2022).

In rice, AC is positively associated with hardness and negatively correlated with viscosity and water absorption High temperatures during grain filling substantially reduce AC by disrupting enzymatic processes required for consistent amylose synthesis (Yamakawa et al., 2007; Zhou et al., 2024). Thus, relatively lower temperatures during the grain-filling period may contribute to the increase in AC in the ratoon season. Furthermore, stubble height modulates these effects: lower stubble height prolongs the growth and grain-filling periods, potentially lowering grain-filling temperatures and thereby increasing AC in RC.

### Mechanism underlying the regulation of ratooning on grain quality

Grain-filling temperatures critically modulate ratoon rice quality. The comparatively lower cumulative temperatures in RC versus MC likely drive observed grain quality variations. This thermal hypothesis is partially supported by our RC–LC comparison, where RC–MC quality patterns were absent (**Figure 5**), though the limited geographical scope and study numbers preclude definitive conclusions. Our findings align with recent multi-latitude analyses and likely reflect, at least in part, temperature effects on ratoon systems. For instance, in a recombinant inbred population of *indica*–*japonica* crosses grown across four regions, cooked rice hardness and stickiness increased with decreasing latitude, while springiness showed the opposite trend (Xu et al., 2019). Kuang et al. (2021) reported that differences in crystal structure and starch thermal properties between RC and MC were closely related to temperature during ripening. High temperature during kernel development can cause spikelet infertility, increase chalkiness, and decrease AC (Peng et al., 2004; Kadam et al., 2014), while lower temperature extends the grain-filling period, resulting in better milling and appearance parameters (Funaba et al., 2006). Recent molecular-level research reported that high temperature enhanced the expression and activity of α-amylase, leading to pitted and uneven starch granule surfaces (Liu et al., 2011).

Significant variation in grain quality traits exists among cultivars grown under identical conditions (Kang et al., 2006; Cameron et al., 2008). Generally, *japonica* varieties are considered to have better ratooning ability than *indica* varieties (Krishanamurthy, 1989). Hybrid varieties show enhanced ratooning ability and higher post-MC harvest dry weight relative to inbred varieties (Chen et al., 2018). Nevertheless, the roles of genetic factors and varietal differences in regulating post-ratooning grain quality remain poorly characterized.

Optimal agronomic practices are crucial to ensuring the productivity and quality of the ratoon rice system, including fertilizer application, planting density, and stubble height. N application at the grain-filling stage of MC was reported to have the most significant effects on tiller sprouting, growth, and yield of ratoon crops (Wang et al., 2021; Yang et al., 2021). However, elevated N fertilization increases endosperm PC, potentially improving grain hardness and milling resistance at the expense of eating and cooking quality (Leesawatwong et al., 2005; Gu et al., 2015; Zhou et al., 2018). Higher planting density enhances interception of photosynthetically active radiation and dry matter accumulation, thereby promoting tiller production, survival, and effective panicle formation (Bozorgi et al., 2011; Zheng et al., 2021). This practice also shows a strong positive correlation with GC but a negative correlation with ASV. Lowering MC stubble height increases ratoon yield by prolonging growth duration, reducing panicle number per unit area, and increasing spikelets per panicle, total spikelets, and leaf area index (Harrell et al., 2009; Yang et al., 2022), which subsequently affects milling parameters and AC.

### Heterogeneity and limitation of our data

While rice ratooning studies have predominantly focused on grain yield, management practices, and varietal breeding, relatively few investigations have addressed grain quality. Quality assessment revealed high methodological standards across the literature, with most studies achieving high-quality scores and demonstrating minimal risk of bias. These findings substantiate the robustness of our analytical conclusions. However, the geographical distribution of research is uneven, with most studies located in Asia. In addition, substantial heterogeneity emerged in our meta-analysis. Rice quality represents a composite trait influenced by both genetic and environmental factors. This multifaceted nature inherently causes grain quality parameters to vary considerably across studies. In West Sumatra, Indonesia, a high-yield perennial rice cropping method called SALIBU involves three cropping cycles, but no grain quality data have been reported (Paiman et al., 2022). Using overwintering (OW) cultivated rice, Liang et al. (2021) reported that four grain quality traits (CRR, ASV, GC, and AC) showed relatively small but significant differences, except for CRR. However, the authors did not trace all cropping seasons, recording only four: RC of 2016, MC of both 2017 and 2018, and RC in 2019. The effects of multiple-harvest rice systems on grain quality require further study.

## Conclusion

Our meta-analysis provides important insights into the benefits of ratooning on grain quality. Compared with MC, RC improved milling properties and appearance traits and altered cooking and sensory quality in a region-dependent manner. This study elucidates the interactive effects of thermal and light factors and agronomic practices on ratoon rice quality.

Future efforts should prioritize synergistic optimization of Genetics × Environment × Management to achieve premium grain quality. Our findings provide novel insights for optimizing ratoon rice production across diverse regions, including the development of region-specific agronomic protocols (e.g., dynamic adjustment of stubble height and planting density) and the screening and breeding of ratoon rice varieties with superior grain quality.

## Data Availability

The original contributions presented in the study are included in the article/**Supplementary Material**. Further inquiries can be directed to the corresponding authors.

## Supplementary material

Supplementary Figure 1 PRISMA diagram for the studies selected and included in the meta-analysis.

Supplementary Figure 2 The risk of bias assessment in the literature. **(A)** A schematic representation of the risk of bias assessment in the literature. Risk of bias was assessed as “+” for low risk, “?” for unclear risk, and “–” for high risk. **(B)** The proportion of three kinds of risk of bias assessments in all the literature.

Supplementary Figure 3 Studies quality assessment. The numbers 1–21 on the left side of the bubble chart represent the 21 questions belonging to the “Quality checklist question.” The histogram shows the score data with two Y-axes. The main Y-axis (left) shows the cumulative scores of all studies in the “Quality checklist question,” and the right Y-axis shows the quality scores of all studies [C(Satisfied)/(C(Satisfied) + C(Dissatisfied)) (count)].

Supplementary Figure 4 Funnel plots of publication bias. All the tests with publication bias (*P* < 0.05) were indicated in red. Black dots represent individual studies, and the gray funnel area represents 95% CIs.
