# Supplementary figures and images for: Meta-analysis reveals that grain quality is improved in ratoon season crop compared with main crop
Source: Front Plant Sci. 2025 Oct 22;16:1604686. doi: 10.3389/fpls.2025.1604686 (PMC12586999; doi:10.3389/fpls.2025.1604686)

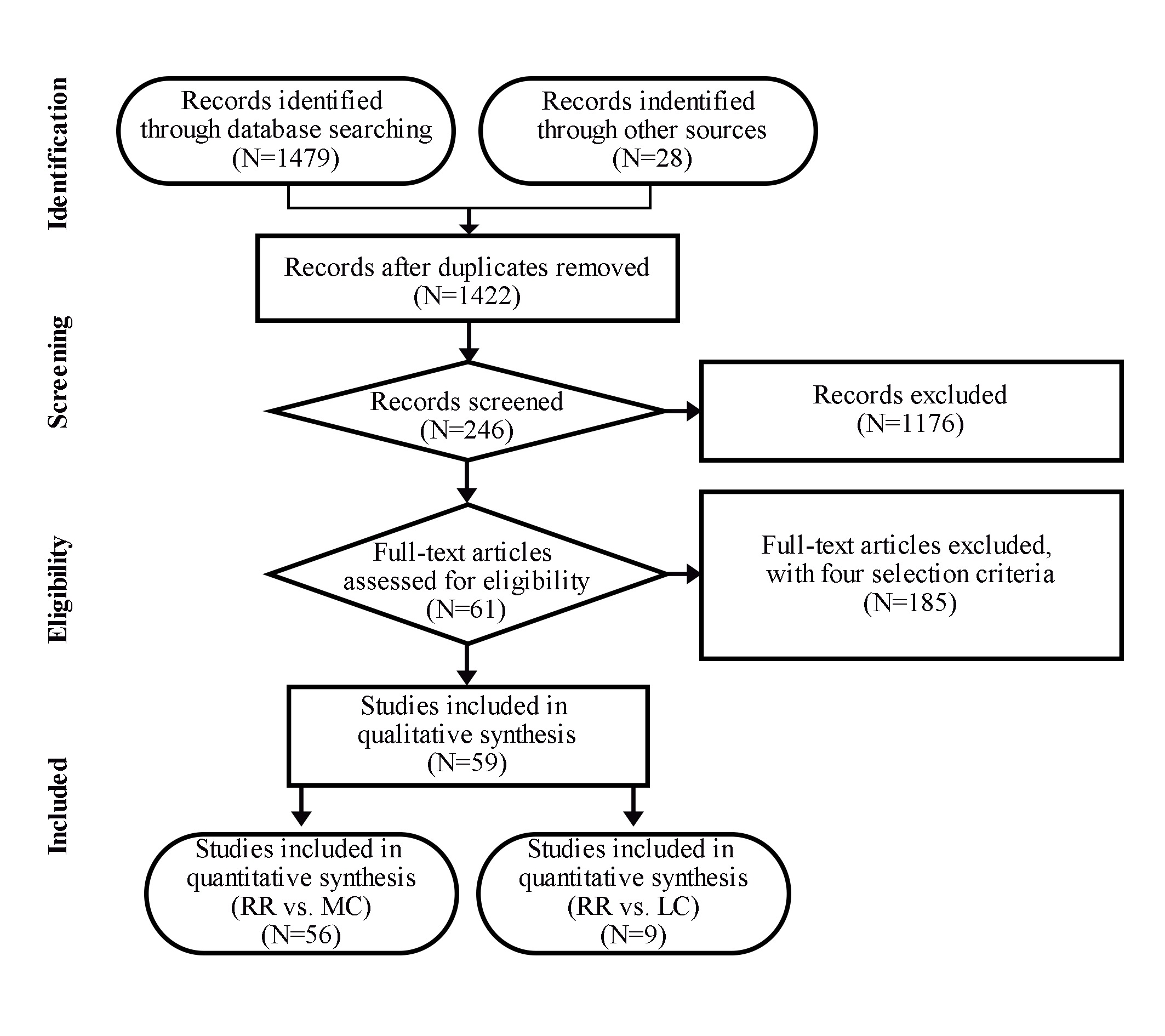

Supplement: Supplementary Figure 1 — PRISMA diagram for the studies selected and included in the meta-analysis. [file Image1.jpeg]

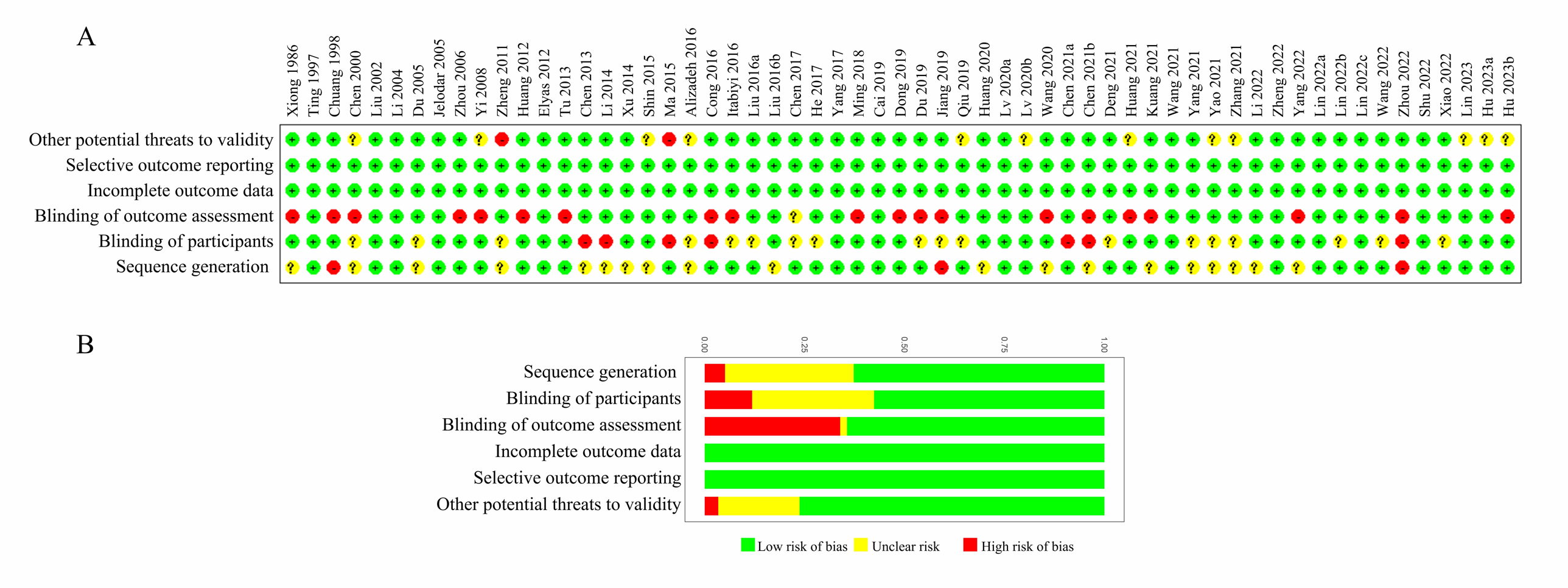

Supplement: Supplementary Figure 2 — The risk of bias assessment in the literature. (A) A schematic representation of the risk of bias assessment in the literature. Risk of bias was assessed as “+” for low risk, “?” for unclear risk, and “–” for high risk. (B) The proportion of three kinds of risk of bias assessments in all the literature. [file Image2.jpeg]

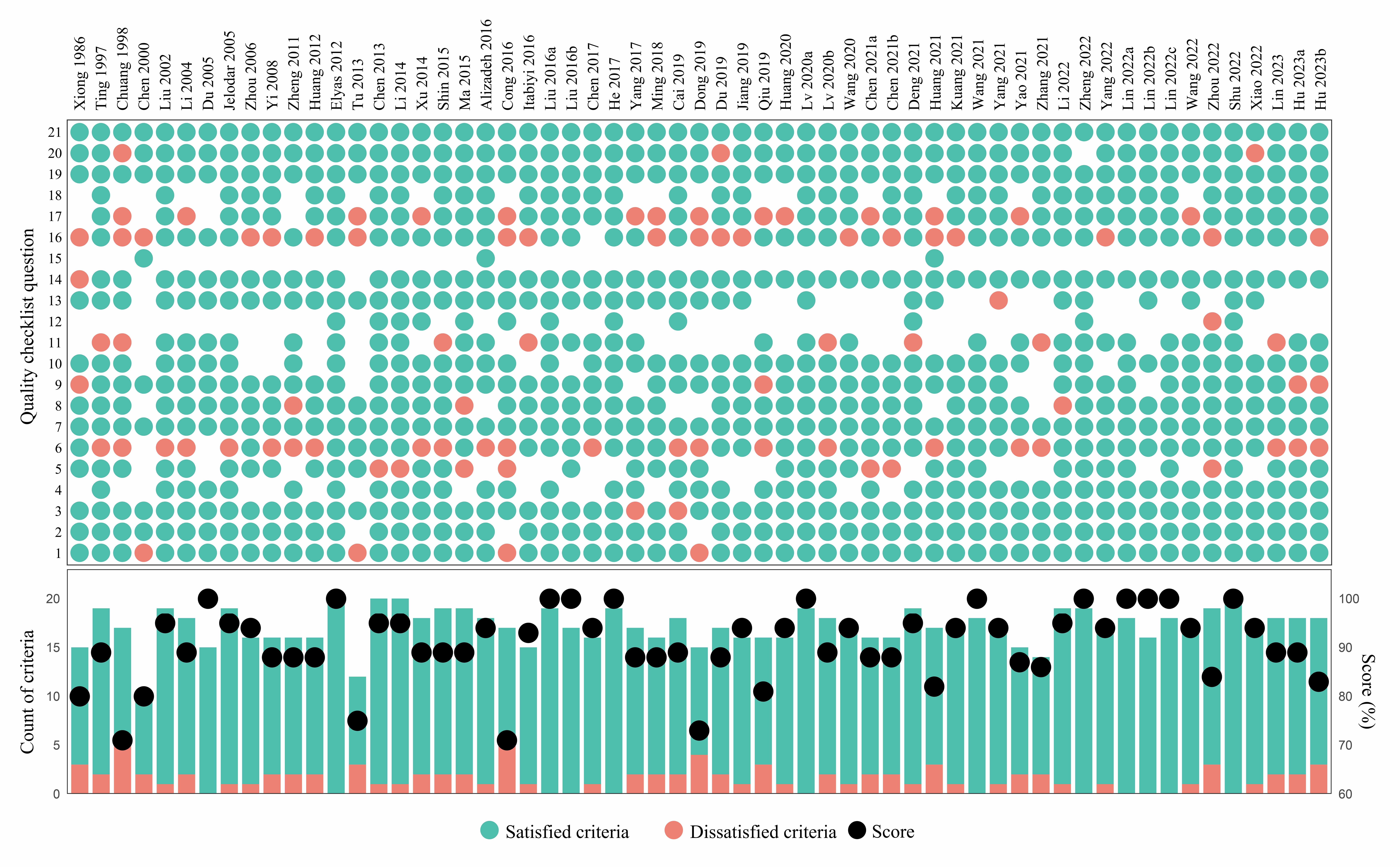

Supplement: Supplementary Figure 3 — Studies quality assessment. The numbers 1–21 on the left side of the bubble chart represent the 21 questions belonging to the “Quality checklist question.” The histogram shows the score data with two Y-axes. The main Y-axis (left) shows the cumulative scores of all studies in the “Quality checklist question,” and the right Y-axis shows the quality scores of all studies [C(Satisfied)/(C(Satisfied) + C(Dissatisfied)) (count)]. [file Image3.jpeg]

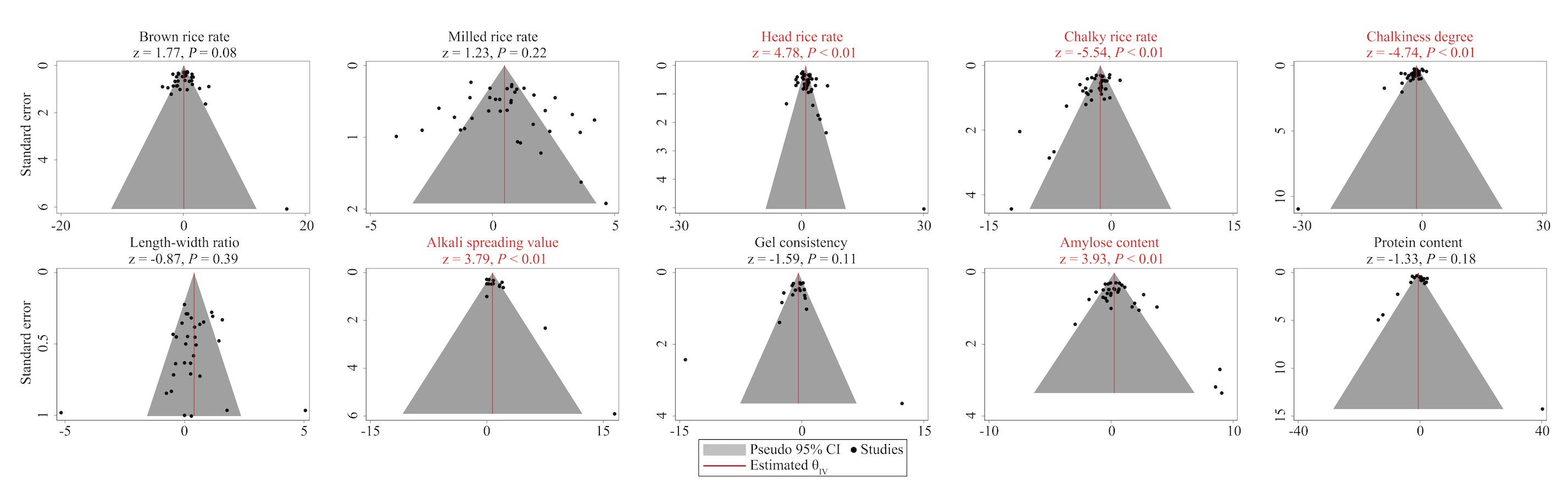

Supplement: Supplementary Figure 4 — Funnel plots of publication bias. All the tests with publication bias (P < 0.05) were indicated in red. Black dots represent individual studies, and the gray funnel area represents 95% CIs. [file Image4.jpeg]
