# Supplementary material for: Meta-analysis reveals that grain quality is improved in ratoon season crop compared with main crop
Source: Front Plant Sci. 2025 Oct 22;16:1604686. doi: 10.3389/fpls.2025.1604686 (PMC12586999; doi:10.3389/fpls.2025.1604686)
Supplement: Supplementary file 7 [file Table3.docx]

**Supplementary Table 1. Q statistic test for subgroup analysis of the categorical factors (RC vs. MC).**

Abbreviations：BRR: Brown rice rate. MRR, milled rice rate. HRR, head rice rate. CRR, chalky rice rate. CD, chalkiness degree. LWR, length-width ratio. ASV, alkali spreading value. GC, gel consistency. AC, amylose content. PC, protein content.

|  |  | **BRR** | **MRR** | **HRR** | **CRR** | **CD** | **LWR** | **ASV** | **GC** | **AC** | **PC** |
| --- | --- | --- | --- | --- | --- | --- | --- | --- | --- | --- | --- |
| *Indica* rice type | ES | 0.17 | 0.79 | 1.61 | -1.93 | -1.86 | 0.41 | 0.93 | -0.61 | 0.41 | -0.57 |
|  | 95%CIs | -0.26,  0.61 | 0.17,  1.41 | 0.99,  2.22 | -2.47,  -1.39 | -2.41,  -1.31 | -0.04,  0.87 | 0.49,  1.37 | -1.07,  -0.14 | -0.03,  0.86 | -1.70,  0.56 |
| *Indica*-*japonica* type | ES | 0.62 | 2.01 | 3.55 | -3.29 | -5.75 | 7.21 | 0.00 | 0.83 | -0.07 | - |
|  | 95%CIs | -0.18,  1.42 | -0.13,  4.16 | 0.72,  6.38 | -4.88,  -1.71 | -8.61,  -2.89 | 3.42,  11.01 | -1.60,  1.60 | -0.84,  2.49 | 1.67,  1.53 | - |
| *Japonica* rice type | ES | -0.37 | -0.11 | 2.01 | -13.50 | -1.54 | -0.05 | 6.52 | 0.13 | -2.12 | 0.35 |
|  | 95%CIs | -2.72,  1.99 | -3.09,  2.87 | -0.50,  4.52 | -40.10,  13.11 | -2.98,  -0.09 | -0.45,  0.35 | 3.04,  10.00 | -1.26,  1.51 | -5.91,  1.68 | -0.57,  1.27 |
|  | Qb | 1.2 | 1.56 | 1.79 | 3.25 | 7.20 | 15.45 | 11.19 | 3.33 | 1.96 | 1.51 |
|  | *P*-value of subgroup | 0.55 | 0.46 | 0.41 | 0.20 | 0.03 | <0.01 | <0.01 | 0.19 | 0.38 | 0.22 |

|  |  | **BRR** | **MRR** | **HRR** | **CRR** | **CD** | **LWR** | **ASV** | **GC** | **AC** | **PC** |
| --- | --- | --- | --- | --- | --- | --- | --- | --- | --- | --- | --- |
| High pile | ES | 0.24 | 0.57 | 1.67 | -1.90 | -2.03 | 0.56 | 0.95 | -0.87 | 0.17 | -0.30 |
|  | 95%CIs | -0.23,  0.72 | -0.03,  1.10 | 0.98,  2.35 | -2.40,  -1.41 | -2.58,  -1.47 | 0.25,  0.87 | 0.49,  1.42 | -2.26,  0.51 | -0.23,  0.57 | -1.64,  1.03 |
| Low pile | ES | 0.16 | 1.82 | 0.92 | -0.82 | -0.91 | -0.84 | 0.00 | -1.25 | 1.55 | 0.17 |
|  | 95%CIs | -0.86,  1.17 | -0.55,  4.19 | 0.42,  1.42 | -2.53,  0.88 | -2.25,  0.43 | -2.74,  1.06 | -0.92,  0.92 | -3.10,  0.61 | 0.40,  2.70 | -1.48,  1.82 |
|  | Qb | 0.02 | 1.02 | 3.01 | 1.42 | 2.27 | 2.04 | 3.27 | 0.10 | 4.93 | 0.19 |
|  | *P*-value of subgroup | 0.88 | 0.31 | 0.08 | 0.23 | 0.13 | 0.15 | 0.07 | 0.75 | 0.03 | 0.66 |

|  |  | **BRR** | **MRR** | **HRR** | **CRR** | **CD** | **LWR** | **ASV** | **GC** | **AC** | **PC** |
| --- | --- | --- | --- | --- | --- | --- | --- | --- | --- | --- | --- |
| South China Plain Hilly | ES | -1.46 | -0.88 | 0.51 | -3.62 | -1.27 | -0.44 | - | - | -0.32 | 0.35 |
|  | 95%CIs | -2.41,  -0.52 | -3.01,  1.24 | -0.48,  1.50 | -6.05,  1.19 | -2.06,  -0.48 | -1.43,  0.56 | - | - | -1.24,  0.60 | -0.57,  1.27 |
| Jiangnan Hilly Plain | ES | -0.14 | 0.66 | 1.61 | -1.66 | -2.19 | -0.05 | 0.43 | -0.04 | 0.12 | 0.12 |
|  | 95%CIs | -0.55,  0.27 | -0.12,  1.43 | 0.52,  2.70 | -2.20,  -1.12 | -3.08,  -1.31 | -0.95,  0.84 | 0.09,  0.77 | -0.31,  0.22 | -0.21,  0.45 | -0.94,  1.19 |
| Middle-lower Yangtze Plain | ES | 0.95 | 1.37 | 1.91 | -2.02 | -1.94 | 0.65 | 1.61 | -0.74 | 1.06 | -12.84 |
|  | 95%CIs | 0.32,  1.57 | 0.66,  2.08 | 1.38,  2.45 | -3.12,  -0.92 | -2.87,  -1.00 | 0.25,  1.05 | 0.80,  2.41 | -1.39,  -0.08 | -0.43,  2.56 | -19.30,  -6.39 |
| Sichuan Basin | ES | 0.51 | -2.19 | 6.71 | -0.40 | -0.61 | 1.18 | 1.67 | -5.53 | 0.95 | -1.19 |
|  | 95%CIs | -1.64,  0.62 | -3.36,  -1.02 | -7.34,  20.76 | -0.90,  0.11 | -1.71,  0.48 | -1.12,  3.48 | 0.65,  2.69 | -13.52,  2.46 | 0.09,  1.81 | -4.80,  2.42 |
| Northern Iran | ES | - | -1.56 | -1.37 | -1.19 | - | -0.76 | 7.53 | -2.44 | 0.18 | -2.48 |
|  | 95%CIs | - | -2.97,  -0.14 | -2.75,  0.01 | -2.92,  0.55 | - | -2.41,  0.90 | 2.98,  12.08 | -4.07,  -0.80 | -3.83,  4.18 | -3.25,  -1.72 |
| South United States | ES | - | -0.88 | 0.30 | - | -1.94 | 0.00 | - | - | - | - |
|  | 95%CIs | - | -1.34,  -0.42 | -0.14,  0.74 | - | -2.47,  -1.41 | -0.44,  0.44 | - | - | - | - |
| South Korea | ES | - | - | 1.68 | - | - | - | - | - | 1.72 | 2.11 |
|  | 95%CIs | - | - | -0.18,  3.53 | - | - | - | - | - | -0.15,  3.60 | 0.11,  4.10 |
| Nigeria | ES | - | - | - | - | - | 0.44 | - | - | - | - |
|  | 95%CIs | - | - | - | - | - | -0.45,  1.32 | - | - | - | - |
|  | Qb | 19.08 | 46.85 | 33.02 | 18.25 | 7.17 | 9.55 | 19.38 | 12.81 | 8.09 | 48.80 |
|  | *P*-value of subgroup | <0.01 | <0.01 | <0.01 | <0.01 | 0.13 | 0.14 | <0.01 | 0.01 | 0.15 | <0.01 |

**Supplementary Table 2. Q statistic test for subgroup analysis of the categorical factors (RC vs. LC).**

|  |  | **BRR** | **MRR** | **HRR** | **CRR** | **CD** | **LWR** |
| --- | --- | --- | --- | --- | --- | --- | --- |
| *Indica* rice type | ES | -1.29 | 0.08 | -0.04 | -1.65 | -0.71 | -0.37 |
|  | 95%CIs | -2.17, -0.42 | -0.77, 0.60 | -0.96, 0.87 | -3.56, 0.27 | -1.91, 0.48 | -0.83, 0.09 |
| *Indica*-*japonica* type | ES | - | - | - | -23.98 | 0.40 | - |
|  | 95%CIs | - | - | - | -40.71, -7.25 | -1.58, 2.38 | - |
| *Japonica* rice type | ES | -0.72 | -0.29 | 2.04 | -8.12 | 3.10 | -1.03 |
|  | 95%CIs | -2.37, 0.93 | -1.90, 1.32 | 0.07, 4.02 | -24.84, 8.60 | -4.71, -10.91 | -2.73, 0.68 |
|  | Qb | 0.36 | 0.05 | 3.53 | 7.28 | 1.66 | 0.53 |
|  | *P*-value of subgroup | 0.55 | 0.81 | 0.06 | 0.03 | 0.44 | 0.47 |

|  |  | **BRR** | **MRR** | **HRR** | **CRR** | **CD** | **LWR** |
| --- | --- | --- | --- | --- | --- | --- | --- |
| South China Plain Hilly | ES | -0.98 | -0.10 | 0.61 | -2.54 | -1.09 | -0.19 |
|  | 95%CIs | -1.69, -0.27 | -0.77, 0.57 | -0.07, 1.29 | -4.02, -1.07 | -2.35, 0.16 | -0.88, 0.51 |
| Jiangnan Hilly Plain | ES | -2.09 | -1.17 | -1.38 | -0.15 | -1.95 | -0.21 |
|  | 95%CIs | -3.50, -0.67 | -3.01, 0.66 | -2.66, -0.10 | -1.03, 0.73 | -3.64, -0.27 | -1.10, 0.67 |
| Middle-lower Yangtze Plain | ES | -0.29 | -0.15 | -0.31 | 0.21 | 0.16 | -0.37 |
|  | 95%CIs | -1.13, 0.55 | -0.69, 0.98 | -1.15, 0.53 | -0.43, 0.86 | -0.68, -0.99 | -1.02, 0.27 |
|  | Qb | 4.76 | 1.65 | 8.03 | 11.28 | 6.11 | 0.17 |
|  | *P*-value of subgroup | 0.09 | 0.44 | 0.02 | <0.01 | 0.05 | 0.92 |

**Supplementary Table 3. R-squared for meta-regression analysis of the continuous factors.**

| **Variate** | **Indicators** | **n of obs** | **Convergence** | **R-squared** | ***P*-value** |
| --- | --- | --- | --- | --- | --- |
| Latitude | BRR | 37 | Yes | 0.3472 | < 0.001 |
|  | MRR | 37 | Yes | 0.0177 | 0.192 |
|  | HRR | 43 | Yes | 0.0000 | 0.777 |
|  | CRR | 39 | Yes | 0.0000 | 0.532 |
|  | CD | 37 | Yes | 0.0000 | 0.911 |
|  | LWR | 31 | Yes | 0.0000 | 0.953 |
|  | ASV | 15 | Yes | 0.6408 | 0.002 |
|  | GC | 20 | Yes | 0.7263 | 0.003 |
|  | AC | 34 | Yes | 0.0000 | 0.359 |
|  | PC | 18 | Yes | 0.0000 | 0.594 |
| Study year | BRR | 44 | Yes | 0.3011 | < 0.001 |
|  | MRR | 45 | Yes | 0.0819 | 0.013 |
|  | HRR | 50 | Yes | 0.0710 | 0.050 |
|  | CRR | 44 | Yes | 0.0012 | 0.101 |
|  | CD | 43 | Yes | 0.0000 | 0.419 |
|  | LWR | 39 | Yes | 0.0553 | 0.066 |
|  | ASV | 16 | Yes | 0.0000 | 0.327 |
|  | GC | 21 | Yes | 0.0000 | 0.315 |
|  | AC | 37 | Yes | 0.0757 | 0.068 |
|  | PC | 21 | Yes | 0.0000 | 0.839 |
| Planting density | BRR | 30 | Yes | 0.0000 | 0.388 |
|  | MRR | 33 | Yes | 0.0336 | 0.075 |
|  | HRR | 37 | Yes | 0.0000 | 0.843 |
|  | CRR | 31 | Yes | 0.0000 | 0.708 |
|  | CD | 29 | Yes | 0.0000 | 0.235 |
|  | LWR | 27 | Yes | 0.0000 | 0.147 |
|  | ASV | 6 | Yes | 1.0000 | < 0.001 |
|  | GC | 15 | Yes | 0.0000 | 0.012 |
|  | AC | 25 | Yes | 0.0000 | 0.920 |
|  | PC | 15 | Yes | 0.0000 | 0.445 |
| N application | BRR | 32 | Yes | 0.0000 | 0.239 |
|  | MRR | 32 | Yes | 0.0000 | 0.765 |
|  | HRR | 36 | Yes | 0.0000 | 0.783 |
|  | CRR | 35 | Yes | 0.0000 | 0.378 |
|  | CD | 34 | Yes | 0.0000 | 0.512 |
|  | LWR | 29 | Yes | 0.0000 | 0.777 |
|  | ASV | 11 | Yes | 0.0000 | 0.925 |
|  | GC | 14 | Yes | 0.0000 | 0.858 |
|  | AC | 22 | Yes | 0.0000 | 0.580 |
|  | PC | 13 | Yes | 0.5004 | 0.012 |
| ΔTavg | BRR | 40 | Yes | 0.1872 | 0.014 |
|  | MRR | 41 | Yes | 0.2371 | 0.001 |
|  | HRR | 46 | Yes | 0.2953 | < 0.001 |
|  | CRR | 43 | Yes | 0.0547 | 0.134 |
|  | CD | 41 | Yes | 0.0337 | 0.062 |
|  | LWR | 38 | Yes | 0.0269 | 0.224 |
|  | ASV | 15 | Yes | 0.0000 | 0.067 |
|  | GC | 19 | Yes | 0.0000 | 0.190 |
|  | AC | 33 | Yes | 0.0000 | 0.490 |
|  | PC | 17 | Yes | 0.0000 | 0.791 |
| ΔPrecipitation | BRR | 37 | Yes | 0.0000 | 0.494 |
|  | MRR | 38 | Yes | 0.0000 | 0.884 |
|  | HRR | 42 | Yes | 0.1219 | 0.015 |
|  | CRR | 40 | Yes | 0.0000 | 0.651 |
|  | CD | 38 | Yes | 0.0000 | 0.509 |
|  | LWR | 36 | Yes | 0.0000 | 0.252 |
|  | ASV | 15 | Yes | 0.0000 | 0.091 |
|  | GC | 19 | Yes | 0.0000 | 0.162 |
|  | AC | 31 | Yes | 0.0000 | 0.289 |
|  | PC | 16 | Yes | 0.0000 | 0.619 |
| Solar radiation | BRR | 43 | Yes | 0.0000 | 0.477 |
|  | MRR | 44 | Yes | 0.0000 | 0.398 |
|  | HRR | 49 | Yes | 0.0000 | 0.569 |
|  | CRR | 43 | Yes | 0.0000 | 0.366 |
|  | CD | 42 | Yes | 0.0000 | 0.419 |
|  | LWR | 38 | Yes | 0.0000 | 0.747 |
|  | ASV | 15 | Yes | 0.0000 | 0.918 |
|  | GC | 20 | Yes | 0.0000 | 0.706 |
|  | AC | 36 | Yes | 0.0000 | 0.395 |
|  | PC | 20 | Yes | 0.0000 | 0.755 |

**Supplementary Table 4. Adjusted estimates after meta trim fill.**

| **Indicators** | **Studies** | **SMD** | **Low** | **High** |
| --- | --- | --- | --- | --- |
| HRR | Observed | 1.407 | 0.897 | 1.917 |
|  | Observed + Imputed | 0.808 | 0.167 | 1.448 |
| CRR | Observed | -1.680 | -2.117 | -1.243 |
|  | Observed + Imputed | -1.527 | -2.041 | -1.013 |
| CD | Observed | -1.778 | -2.266 | -1.289 |
|  | Observed + Imputed | -1.460 | -2.064 | -0.857 |
| ASV | Observed | 0.897 | 0.483 | 1.312 |
|  | Observed + Imputed | 0.523 | 0.007 | 1.040 |
| AC | Observed | 0.388 | 0.018 | 0.758 |
|  | Observed + Imputed | 0.364 | -0.009 | 0.738 |
